# Effects of Skin Friction on Tactile P300 Brain-Computer Interface Performance

**DOI:** 10.1155/2021/6694310

**Published:** 2021-02-09

**Authors:** Ying Mao, Jing Jin, Shurui Li, Yangyang Miao, Andrzej Cichocki

**Affiliations:** ^1^Key Laboratory of Advanced Control and Optimization for Chemical Processes, Ministry of Education, East China University of Science and Technology, Shanghai, China; ^2^Skolkovo Institute of Science and Technology (SKOLTECH), Moscow 143026, Russia; ^3^Nicolaus Copernicus University (UMK), Torun, Poland

## Abstract

Tactile perception, the primary sensing channel of the tactile brain-computer interface (BCI), is a complicated process. Skin friction plays a vital role in tactile perception. This study aimed to examine the effects of skin friction on tactile P300 BCI performance. Two kinds of oddball paradigms were designed, silk-stim paradigm (SSP) and linen-stim paradigm (LSP), in which silk and linen were wrapped on target vibration motors, respectively. In both paradigms, the disturbance vibrators were wrapped in cotton. The experimental results showed that LSP could induce stronger event-related potentials (ERPs) and achieved a higher classification accuracy and information transfer rate (ITR) compared with SSP. The findings indicate that high skin friction can achieve high performance in tactile BCI. This work provides a novel research direction and constitutes a viable basis for the future tactile P300 BCI, which may benefit patients with visual impairments.

## 1. Introduction

Brain-computer interface (BCI) is a new communication and control technique that establishes the interaction between the human brain and external devices without the involvement of normal neural pathways. Thus far, BCI has shown great application values in medical rehabilitation (such as stroke patients [1] and amyotrophic lateral sclerosis (ALS) [2]), auxiliary control (such as computer typing [3], cursor control [4], and wheelchair navigation [5]), and life entertainment (such as game interaction [6] and smart home [7]). The commonly used brain activity patterns in current BCI systems include motor imagery (MI) [8–11], steady-state visual evoked potentials (SSVEP) [12, 13], and event-related potentials (ERPs) [14, 15].

P300 potential, a commonly used component in ERP, is usually related to psychological and cognitive functions and is generally induced by the oddball paradigm, in which the occurrence of the target stimulus is a small-probability event [16]. It appears within 250–500 ms after the stimulus occurs and has an evident positive amplitude [17]. The early P300-based BCIs were primarily evoked by visual stimuli. For example, the most classic visual P300-based BCI was introduced by Farwell and Donchin in 1988 [18]. They used a 6 × 6 matrix presenting 26 letters and 10 digits to provide visual stimuli. The subjects were informed to gaze at the target character and silently count the number of flashes of the target character. Afterward, Hill et al. [19] first proposed an auditory-based BCI that allows the subject to make a binary decision with a spatial informative cue. However, visual and auditory BCIs are inapplicable to patients with vision or hearing impairments. Therefore, studies should focus on the development of tactile-based BCI for patients with vision and hearing impairments. The first tactile-based BCI system was introduced by Brouwer and Van Erp [20]. They placed vibrotactile stimulators on different locations around the subject's waist and instructed the subject to focus on the designated vibrator to complete the task of target selection. Their study demonstrated the effectiveness of tactile-based BCI. Following their work, researchers have attempted to apply tactile stimulation to other parts of the body, such as the head [21], fingers [22], chest [23], back [24], and cheeks [25]. In addition, studies have been conducted on the influence of factors, such as the number of tactile stimuli [26], stimulus frequency [27], and stimulus onset asynchrony [20], on the tactile BCI system. Meanwhile, the tactile BCI system has been verified on patients, and several research results have been achieved. Kaufmann et al. [28] developed four-choice BCI paradigms based on different modalities for a locked-in syndrome patient. Their results demonstrated that the tactile modality outperformed other modalities (visual and auditory). Silvoni et al. [29] investigated the tactile ERP and classification accuracy in a sample of ALS patients and found no significant differences in the accuracy between ALS patients and healthy participants, proving the feasibility of tactile BCI for patients with diseases. Most recently, Murovec et al. [30] demonstrated the potential of communicating by a tactile BCI system for patients with disorders of consciousness.

Tactile sensing is a comprehensive sensation formed by the receptor response to skin stimulation [31]. As the main sensing channel of tactile BCI, tactile perception is a complicated process that is closely related to the surface characteristics of the contact [32]. Friction plays an important role in tactile perception, and the deformations produced by friction can stimulate sensory receptors in the skin when the skin scans the surface of fabrics [33, 34]. ERP technique has been applied to investigate the relationship between fiction and tactile perception. ERPs evoked by fingertip friction are affected by the surface texture and friction coefficient, and the response sensibility has a strong relation to the frictional stimulus [35].

However, no research has been conducted on the effect of skin friction on tactile BCI. In this study, we investigated the effects of skin friction on tactile BCI. Oddball paradigm was adopted to design our experiments, and five vibrators were fixed on the subject's left palm, right palm, abdomen, left ankle, and right ankle. Based on the fact that different fabrics provide varying rough senses, we designed two paradigms in this study, namely, the silk-stim paradigm (SSP) and linen-stim paradigm (LSP). We also compared the ERP features induced by the two paradigms and their BCI performance.

## 2. Materials and Methods

### 2.1. Subjects

Ten right-handed healthy subjects, including five males and five females (labeled S1–S10), aged 23–28 years (mean 24.7 ± 1.42), participated in our experiments. All subjects have intact tactile sensation and had no history of tactile sensation impairment (self-reported). Before each experiment, the subjects initially experienced the vibration of the vibrators and then we started the experiment only when the subjects reported that they could recognize the vibrations from each location. The experiments were given permission from the Shanghai Xuhui Central Hospital Ethics Committee on July 10, 2020, and each subject signed a written consent before the experiment. Before each experiment, we gave the subjects a detailed description of the task and told them that they could terminate at any time if they felt any discomfort during the experiment.

### 2.2. Stimulus Generation and Data Recording

**Figure 1:**
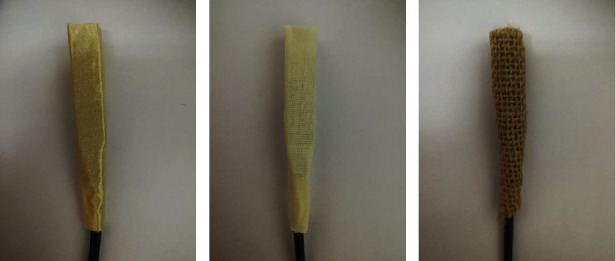
Stimulators with different fabrics: (a) silk, (b) cotton, and (c) linen.

In this paper, tactile stimuli were provided by vibrotactile stimulators (g.VIBROstims) driven by a g.STIMbox (g.tec medical engineering GmbH, Austria). The vibration motors were wrapped in different fabrics to give the subjects different rough senses. We used fabrics to provide different tactile sensations because they are one of the most common skin-touch materials. The fabrics were selected to enlarge their differences and to ensure that the subjects can distinctly perceive the difference between them. The fabrics used in this study were silk (Figure 1(a)), cotton (Figure 1(b)), and linen (Figure 1(c)).  Table 1 shows the yarn count, warp-weft density, and surface density of the fabrics. The stimulus duration and the interval between stimuli were, respectively, set at 200 and 400 ms [36].

**Figure 2:**
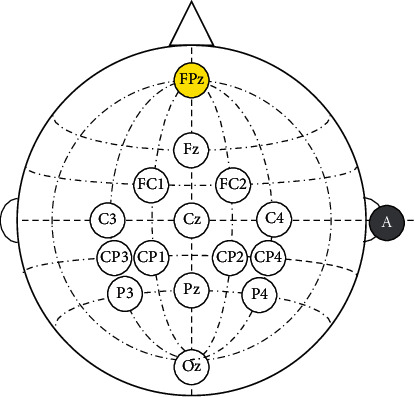
The configuration of the selected electrode positions from the 10–20 system.

Electroencephalography (EEG) signals were collected by an electrode cap (g.EEGcap) and filtered and amplified by an EEG amplifier (g.USBamp). The amplifier was set with a sampling rate of 256 Hz, a band-pass filter from 0.1 to 30 Hz and a notch filter of 50 Hz. Fourteen channels selected in accordance with the international 10–20 electrode placement system were Fz, FC1, FC2, C3, Cz, C4, CP3, CP1, CP2, CP4, P3, Pz, P4, and Oz (Figure 2). The reference and ground electrodes were placed at the forehead (FPz) and the right mastoid (A). The electrode impedances were kept below 10 kΩ during the experiment.

### 2.3. Experimental Design

**Figure 3:**
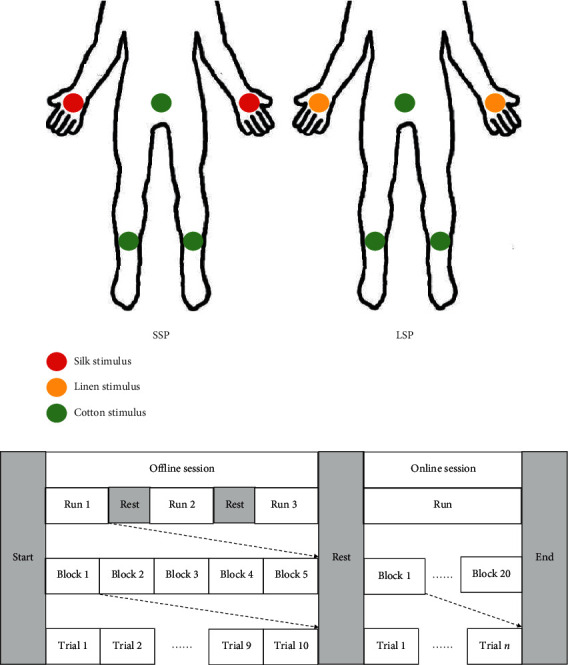
Experimental design. (a) Two paradigm designs; (b) flowchart of the experiment.

We adopted the commonly used oddball paradigm for the survey. We fixed five vibrators on the subject's left palm, right palm, abdomen, left ankle, and right ankle. During the experiments, only the stimulation delivered to the left or right palm could be designated as the target, whereas the others were regarded as disturbance stimuli that were intended to reduce the probability of the target stimulus. As in [37], the lower the probability of a target stimulus in a small-probability event, the greater the amplitude of the induced P300 signal. Two paradigms were designed in this study (Figure 3(a)), namely, SSP and LSP, in which silk and linen were wrapped on the target vibration motors, respectively. To maintain the consistency of the disturbance stimulus, we selected cotton with a roughness between that of silk and linen as the disturbance stimulus of the two paradigms. Therefore, in both paradigms, the disturbance vibrators were wrapped in cotton.

During the experiment, the subjects were instructed to sit relaxed in a chair and put their hands on the left and right arms of the chair. They were asked to keep their eyes straight ahead and not to look at their stimulated body locations. The order of the two paradigm experiments was randomized for each subject. For each paradigm, the subject was instructed to perform the offline session to train and build a classification model and then perform the online session to test the performance of the current paradigm.  Figure 3(b) shows the procedure of the experimental protocol. The offline session contained three runs, and each run included five blocks. In each block, the stimulator on the left or right palm was selected as the target and to be focused on. The target in one block was the same and was indicated to the subject by a vibration cue of 2 s at the beginning of each block. One block consisted of 10 trials. In each trial, the five vibrators, of which one was the target, and the other four were nontargets, vibrated once in a pseudorandom order without repetition. The subject was instructed to concentrate on the target stimulus by covertly counting the number of target vibrations. Between two runs, the subjects had a 5 min break. After three runs of the offline session, the classification model could be built based on the offline data. As for the online session, 20 blocks were included (each type of target was tested 10 times), but the number of trials in each block was varied. The online real-time feedback could be presented every time a block recognition was completed. When recognizing classification results for each block, the adaptive strategy developed in our earlier work was applied [38]. The system outputted the result when two successive results were the same in each block recognition, thereby greatly reducing the number of trials and time.

### 2.4. Feature Extraction and Classification

We selected 100 ms prestimulus and 800 ms poststimulus data segments (900 ms in total) based on the labels we marked during the experiment to perform feature extraction and classification. The data of 100 ms before stimulus onset were used for baseline correction of the data afterward. A third-order Butterworth filter with a band-pass from 0.1 Hz to 30 Hz was applied to filter the raw EEG data. To reduce the large-amplitude outliers in the EEG caused by eye blinks, eye movements, muscle activities, or subject movements, we applied the Winsorizing method to filter artifacts with amplitudes less than 10 percentiles and more than 90 percentiles of the amplitude distribution [39]. Then, the data were downsampled from 256 Hz to 36 Hz by selecting every 7^th^ sample to eliminate the curse of dimensionality. Therefore, the feature vector of 14 × 29 (14 channels and 29 sample points) was available for the classifier.

The classification algorithm is the core of a BCI system. A simple and effective algorithm that has often been used in P300-based BCI systems is Fisher's linear discriminant analysis (FLDA) [40, 41]. FLDA is a benchmark method to determine the optimal separation hyperplane between two classes [42]. However, as the number of input features increases, the classification performance of FLDA will deteriorate if the training sample is insufficient [43]. Stepwise linear discriminant analysis (SWLDA) is an extension of FLDA, which reduces the feature space by selecting appropriate features [44]. SWLDA has the advantage of automatic feature extraction, but it cannot guarantee convergence of the model [43]. Another widely used linear classification algorithm is the Bayesian linear discriminant analysis (BLDA) [39], which combines the FLDA algorithm and Bayesian regression iteration. It can solve the problem of data overfitting effectively. As an effective classification algorithm, BLDA has been applied in many P300-based BCI studies [45, 46]. Therefore, we applied the BLDA algorithm in this study. The implementation of BLDA is extremely simple because it is completely automatic and does not require user intervention to adjust hyperparameters. The basic BLDA classification rule is as follows:

(1) $$\begin{array}{c} m=\beta {\left(\beta XX^{T}+I^{\prime }\left(\alpha \right)\right)}^{-1}Xt, \end{array}$$

where *X* denotes the horizontal stacking matrix of feature vectors, *t* denotes a vector that contains the regression targets, and *m* denotes the weight vector of the BLDA classifier and depends on the hyperparameters *α* and *β*. Hyperparameters *α* and *β* can be estimated automatically and iteratively through the evidence process. *K* is the number of features and *I*′(*α*) is a *K*+1 dimensional diagonal matrix.

For a new input vector $\hat{x}$,

(2) $$\begin{array}{c} \mu =m^{T}\hat{x}, \end{array}$$

where *μ* can be used to output decisions in the P300-based BCI. In our study, when performing online classification, five feature vectors from five stimulus positions were inputted into the classifier to calculate the probability that they belong to the target. Then, the one with the largest probability was identified as the classification result.

### 2.5. Data Analysis and Statistics

In this study, we adopted classification accuracy (ACC) and information transfer rate (ITR) to evaluate the BCI performance of our paradigms. The ACC was defined as the ratio of the number of targets correctly classified by the classifier to the total number of targets tested. ITR indicates the number of bits the system can transmit per minute, and it can be evaluated as follows [20]:

(3) $$\begin{array}{c} B=\log_{2}\;N+\text{Acc}\cdot \log_{2}\;N+\left(1-\text{Acc}\right)\cdot \log_{2}\left(\frac{1-\text{Acc}}{N-1}\right),\\ \text{ITR}=B\cdot \frac{60}{T}, \end{array}$$

where *N* denotes the number of possible decisions and *T* represents the time of a trial. In this work, the value of *N* was 5.

We adopted statistical analysis to investigate the statistical differences between the two paradigms across all subjects. We considered using nonparametric statistical methods for analysis due to the relatively small sample size (*n* = 10). The Wilcoxon signed-rank test was used to estimate the statistical difference between the two proposed paradigms. A *p* value less than 0.05 could be regarded as a significant difference. The effect size was calculated as Cohen's *d* for the results of the Wilcoxon signed-rank test. SPSS software was applied to perform the Wilcoxon signed-rank test and G^*∗*^Power software was applied to calculate the effect size and statistical power of the Wilcoxon signed-rank test.

## 3. Results

### 3.1. ERPs

**Figure 4:**
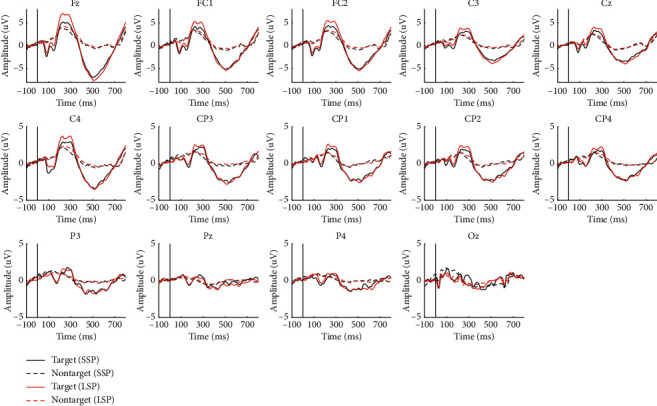
Grand averaged ERP responses of the two paradigms across 10 subjects from 100 ms before the stimulus onset to 800 ms after over 14 electrodes.

Figure 4 shows the grand averaged ERPs from 100 ms before the stimulus onset to 800 ms after for 10 subjects over 14 electrodes in the two paradigms. In the figure, the solid lines are the responses to the target stimulus, and the dashed ones are to the nontarget. Similar ERP components were elicited in both paradigms, but no obvious difference was observed in the ERP amplitude between the two paradigms from the figure.

**Figure 5:**
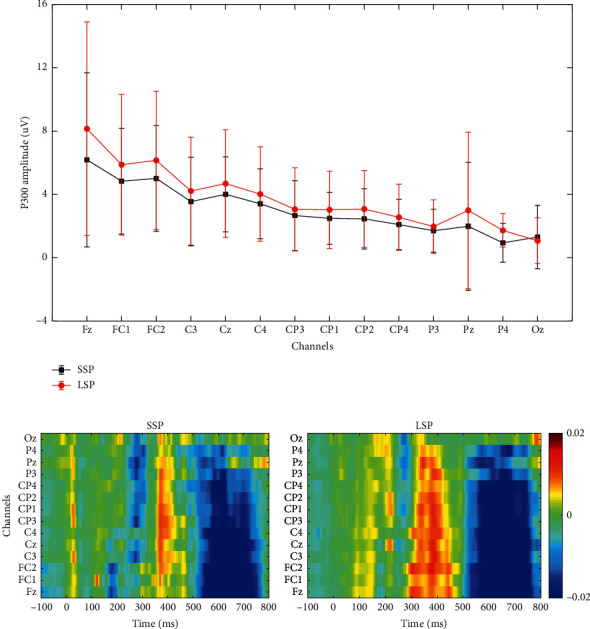
(a) P300 amplitudes over different electrodes of two paradigms across 10 subjects and (b) signed r-square value maps from 100 ms before the stimulus onset to 800 ms after for 10 subjects over 14 electrodes of the two paradigms.

Figure 5(a) illustrates the mean P300 amplitude at different electrodes over 10 subjects. To calculate the amplitude of the P300, we searched for the peak value of the EEG data between 250 and 500 ms after the target stimulus onset. P300 amplitude was defined as the average of ERP amplitudes at the peak point ±25 ms [47]. Notably, the P300 amplitudes of all electrodes in LSP were significantly larger than those in SSP (except for Oz). To observe the discrimination between the target and nontarget in the two paradigms, we adopted r-squared values to quantify the differences between targets and nontargets. The data segment from 100 ms before the stimulus onset to 800 ms after was extracted, and the target and nontarget ERPs were separated as the input of the following calculation to obtain the r-squared values:

(4) $$\begin{array}{c} r^{2}=\left(\frac{\sqrt{N_{1}N_{2}}}{N_{1}+N_{2}}\cdot \frac{\text{mean}\left(x|y=1\right)-\text{mean}\left(x|y=0\right)}{\text{std}\left(x|y=1,0\right)}\right), \end{array}$$

where *N*_1_ and *N*_2_ denote the numbers of features of each class (target and nontarget, resp.). *y* is the class label (“1” stands for the target samples and “0” for the nontarget ones) and *x* represents the value of the sample.  Figure 5(b) shows the signed r-squared value maps from 100 ms before the stimulus onset to 800 ms after for 10 subjects over 14 electrodes under the two paradigms. In the figure, the darker the color, the more evident the discrimination between the two classes. The figure also shows that the difference between targets and nontargets in the LSP condition was larger than that in the SSP condition, especially for P300 components.

### 3.2. Offline and Online BCI Performance

**Figure 6:**
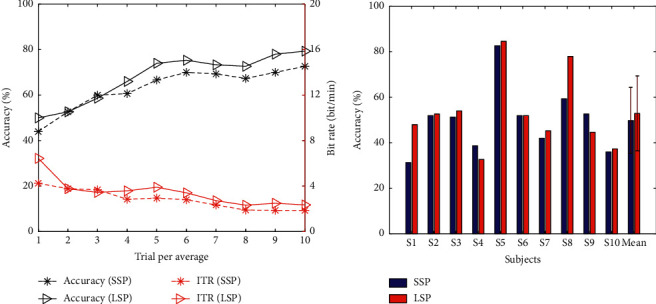
Offline performances of two paradigms. (a) Accuracy and raw bit rate overlapping over 1–10 trials averaged by 10 subjects. (b) Single-trial accuracy for each subject.

Figure 6 shows the offline performances of the two paradigms.  Figure 6(a) depicts the offline accuracy and raw bit rate averaged by 10 subjects, which were calculated by 15-fold cross-validation, across 1–10 trials. It can be seen from the figure that the LSP yielded better offline performances, that is, higher ACC and ITR, than SSP.  Figure 6(b) presents the single-trial offline ACC of the 10 subjects for the two paradigms. From the figure, the single-trial accuracy of LSP was a bit higher than that of SSP, but no significant differences were found.

**Figure 7:**
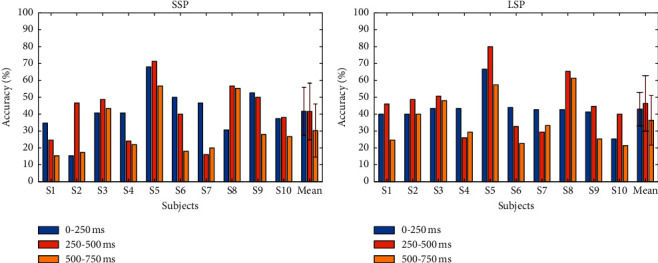
Contribution of different time windows to the offline accuracy for each subject in the two paradigms.

Figure 7 shows the contribution of different time windows to offline accuracy. The figure depicts the offline accuracy of each participant for different 250 ms epochs before the P300 (0–250 ms from stimulus onset), during (250–500 ms), and after (500–750 ms). The results indicate that the early and middle epochs played a pivotal role in offline classification in both paradigms.

Table 2 lists the online performance of the 10 subjects in detail. We applied three indexes, that is, ACC, ITR, and the average number of trials (AVT) to describe the online performance. The table also shows the results of the Wilcoxon signed-rank test. From the table, the ACC and ITR of the LSP were significantly higher than those of SSP (*p* < 0.05), whereas no significant difference was found in the AVT between the two paradigms.

## 4. Discussion

Our study primarily aimed to investigate the effects of skin friction on tactile BCI. Fabrics with different roughness were adopted to produce different tactile sensing. We compared the ERP features and BCI performance of two paradigms, namely, SSP and LSP, in which stimulators wrapped with silk or linen, respectively, were designated as targets. In both paradigms, stimulators wrapped with cotton were referred to as the disturbance stimuli. The offline and online results indicate that the LSP obtained higher classification accuracy and ITR than the SSP.

Previous researches have demonstrated that the tactile oddball paradigm can induce recognizable P300 components [20, 25]. We have found similar P300 components in both paradigms in our study (Figure 4). The postcentral gyrus is responsible for the systematic integration of bilateral body parts and somatic information, and the response of the brain to tactile stimulation is generally concentrated in the central and posterior gyri [48].  Figure 5(b) shows that P300 components were induced on the postcentral gyrus in both paradigms, whereas the P300 feature difference between the target and nontargets in the LSP condition was more evident than that in SSP. As is indicated in a previous study, the late positive component (LPC) of ERP is affected by skin friction, and high fiction leads to high LPC amplitudes [49]. Similar findings were found in this study. Figure 5(a) illustrates that the P300 amplitudes of all electrodes in the LSP condition were larger than those in the SSP condition. Our findings confirm that the amplitude of brain signals response (ERP potentials) is affected by the skin friction coefficient, and high friction can induce large P300 amplitudes. From Figure 4, we can also find an obvious negative wave (N1) around 150 ms, which reflects the activity of the occipitotemporal cortex around 150–250 ms after stimulation [50]. The N1 ERP reflects early attention allocation, thus facilitating further perceptual processing and classification of stimuli [51]. It can be seen that the SSP induced stronger N1 than LSP; in other words, the SSP might evoke stronger attention processes preceding efficient stimulus classification. The reason may be that the vibration intensity of the linen-wrapped vibrator was slightly weaker than that of the silk-wrapped vibrator. However, from Figure 7, the difference in N1 did not have a great influence on the classification.

In terms of offline classification accuracy and information transfer rate (Figure 6(a)), the LSP showed a higher classification and ITR after three trials compared with SSP. From Figure 6(b), the single-trial accuracy showed no significant difference between the two paradigms. The results indicate that the effect of skin friction on tactile BCI performance was based on a large number of trials for average, and the advantage of high friction became evident as the number of trials gradually increased. As for the online performance, the results listed in Table 2 indicate that the LSP achieved a significantly higher online classification accuracy and information transfer rate than the SSP (*p* < 0.05). Thus, the LSP achieved higher performance than the SSP, and it may be inferred that high skin friction can achieve a high tactile BCI performance. Tactile sensing is a complex sensation and different subjects have different sensitivity to the roughness of the contact [31]. Therefore, for some subjects with low sensitivity to roughness, such as S4, S6, and S9 (see Figure 6(b)), the difference between different fabrics may not be considerable.

Our findings confirm the correlation between the tactile BCI system and skin friction, which can be beneficial to studies on haptics, tactile stimulation materials, and bionic skin. However, several limitations existed in our research. Considering that only healthy subjects participated in our experiments, whether the same conclusion can be reached for the disabled is uncertain. Therefore, in future studies, we will further verify the effect of skin friction on patients, such as those with ALS, stroke, and disorders of consciousness.

## 5. Conclusions

In the present work, we explored the influence of different skin frictions on the tactile-based BCI system. The results demonstrate that the LSP yielded better classification accuracy and ITR compared with the SSP. The findings of the ERP analysis indicated that the P300 amplitude was affected by friction. High friction will lead to a large P300 amplitude. Future work should focus on the effect of other surface properties of stimulators, such as bumps and surface texture, on tactile BCI performance.

## Figures and Tables

**Table 1:** Yarn counts, warp-weft density, and surface density of fabric samples.

Samples	Yarn counts (Tex)	Warp-weft density (/10 cm)	Surface density (g/m^2^)
Silk	2.4	630 × 490	80
Cotton	14.6	153 × 80	126
Linen	27.8	58 × 72	160

**Table 2:** Online classification performance.

Subjects	ACC (%)		ITR (bit/min)		AVT	
	SSP	LSP	SSP	LSP	SSP	LSP
S1	50.00	70.00	2.08	5.09	3.10	3.30
S2	70.00	75.00	4.67	5.69	3.60	3.55
S3	75.00	95.00	5.86	10.90	3.45	3.55
S4	50.00	70.00	1.89	4.26	3.40	3.95
S5	95.00	100.00	12.10	15.23	3.20	3.05
S6	60.00	60.00	3.44	3.39	3.20	3.25
S7	50.00	55.00	1.76	2.23	3.65	3.85
S8	90.00	100.00	9.72	14.07	3.40	3.30
S9	50.00	55.00	2.01	2.52	3.20	3.40
S10	55.00	75.00	2.56	6.13	3.35	3.30
AVG ± STD	64.50 ± 17.23	75.50 ± 17.39	4.61 ± 3.62	6.95 ± 4.74	3.36 ± 0.18	3.45 ± 0.28
*p* value	0.007		0.007		0.183	
Effect size (*d*)	1.355		1.320		0.435	
Statistical power (1-*β*)	0.969		0.960		0.271	

ACC = classification accuracy, ITR = information transfer rate, AVT = average number of trials, AVG = average, STD = standard deviation, SSP = silk-stim paradigm, LSP = linen-stim paradigm.

## Data Availability

The data used to support the findings of this study are available from the corresponding author upon request.
